# Dysregulation of systemic soluble immune checkpoints in early breast cancer is attenuated following administration of neoadjuvant chemotherapy and is associated with recovery of CD27, CD28, CD40, CD80, ICOS and GITR and substantially increased levels of PD-L1, LAG-3 and TIM-3

**DOI:** 10.3389/fonc.2023.1097309

**Published:** 2023-03-30

**Authors:** Bernardo L. Rapoport, Helen C. Steel, Carol A. Benn, Simon Nayler, Teresa Smit, Liezl Heyman, Annette J. Theron, Nomsa Hlatshwayo, Luyanda L.I. Kwofie, Pieter W.A. Meyer, Ronald Anderson

**Affiliations:** ^1^ Department of Immunology, Faculty of Health Sciences, University of Pretoria, Pretoria, South Africa; ^2^ Medical Oncology Centre of Rosebank, Johannesburg, South Africa; ^3^ Netcare Breast Care Centre, Johannesburg, South Africa; ^4^ Department of Surgery, Faculty of Health Sciences, University of the Witwatersrand, Johannesburg, South Africa; ^5^ Drs Gritzman & Thatcher Inc. Laboratories, University of the Witwatersrand Donald Gordon Medical Centre, Johannesburg, South Africa; ^6^ Department of Immunology, Tshwane Academic Division of the National Health Laboratory Service, Pretoria, South Africa

**Keywords:** breast cancer, co-inhibitory checkpoints, co-stimulatory checkpoints, cytotoxic T cells, immunotherapy, immune dysregulation, neoadjuvant chemotherapy

## Abstract

Neoadjuvant chemotherapy (NAC) may alter the immune landscape of patients with early breast cancer (BC), potentially setting the scene for more effective implementation of checkpoint-targeted immunotherapy. This issue has been investigated in the current study in which alterations in the plasma concentrations of 16 soluble co-stimulatory and co-inhibitory, immune checkpoints were measured sequentially in a cohort of newly diagnosed, early BC patients (n=72), pre-treatment, post-NAC and post-surgery using a Multiplex^®^ bead array platform. Relative to a group of healthy control subjects (n=45), the median pre-treatment levels of five co-stimulatory (CD27, CD40, GITRL, ICOS, GITR) and three co-inhibitory (TIM-3, CTLA-4, PD-L1) soluble checkpoints were significantly lower in the BC patients vs. controls (*p*<0.021-*p*<0.0001; and *p*<0.008-*p*<0.00001, respectively). Following NAC, the plasma levels of six soluble co-stimulatory checkpoints (CD28, CD40, ICOS, CD27, CD80, GITR), all involved in activation of CD8^+^ cytotoxic T cells, were significantly increased (*p*<0.04-*p*<0.00001), comparable with control values and remained at these levels post-surgery. Of the soluble co-inhibitory checkpoints, three (LAG-3, PD-L1, TIM-3) increased significantly post-NAC, reaching levels significantly greater than those of the control group. PD-1 remained unchanged, while BTLA and CTLA-4 decreased significantly (*p*<0.03 and *p*<0.00001, respectively). Normalization of soluble co-stimulatory immune checkpoints is seemingly indicative of reversal of systemic immune dysregulation following administration of NAC in early BC, while recovery of immune homeostasis may explain the increased levels of several negative checkpoint proteins, albeit with the exceptions of CTLA-4 and PD-1. Although a pathological complete response (pCR) was documented in 61% of patients (mostly triple-negative BC), surprisingly, none of the soluble immune checkpoints correlated with the pCR, either pre-treatment or post-NAC. Nevertheless, in the case of the co-stimulatory ICMs, these novel findings are indicative of the immune-restorative potential of NAC in early BC, while in the case of the co-inhibitory ICMs, elevated levels of soluble PD-L1, LAG-3 and TIM-3 post-NAC underscore the augmentative immunotherapeutic promise of targeting these molecules, either individually or in combination, as a strategy, which may contribute to the improved management of early BC.

## Introduction

Notwithstanding the existence of profound immune dysregulation in advanced metastatic breast cancer (BC), it is now well recognized that early disease is also associated with both localized and systemic immune dysfunction (1–4). In the tumor microenvironment (TME), macrophages of the M2-like immunosuppressive phenotype appear to predominate (5–7), where they effectively exclude cytotoxic CD8^+^ T cells from reaching their tumor targets (8). In the case of systemic immunosuppression in early BC, prominent mechanisms include decreased numbers and/or activation of conventional dendritic cells (1, 3, 4), as well as increased numbers of monocytes with an M2-like phenotype (6, 9).

Recently, soluble co-inhibitory immune checkpoint molecules (ICMs) have been implicated as being potential mediators of the systemic immune dysregulation, which is associated with certain types of malignancy (10, 11). Prominent among these soluble co-inhibitory ICMs are: i) cytotoxic T-lymphocyte-associated protein (CTLA-4); ii) programmed cell death protein 1 (PD-1) and its ligand, PD-L1; iii) lymphocyte activation gene 3 (LAG-3); and iv) T cell immunoglobulin and mucin-domain-containing protein 3 (TIM-3) (12). On the other hand, less is known about alterations in the systemic levels of co-stimulatory ICMs measured prior to and following treatment of various types of malignancy. Although the origins of soluble systemic ICMs remain unclear, leakage from the TME seems plausible. In this setting, soluble co-inhibitory ICMs may originate from tumor cells *per se*, as well as from structural cells such as cancer-associated fibroblasts and cells of the innate and adaptive immune systems recruited to the TME (13, 14). Soluble variant isoforms of the ICMs, which lack transmembrane domains, may arise from alternative RNA splicing, or, alternatively, by proteolytic detachment from cell membranes (10, 12). Importantly, soluble co-inhibitory ICMs appear to retain their biological activities (10, 12).

In the setting of early BC, neoadjuvant chemotherapy (NAC) preceding surgical excision of residual tumor tissue remains a cornerstone of the treatment of this malignancy and strategies to improve the clinical efficacy and safety of this pre-operative procedure are ongoing, including evaluation of the combination of co-inhibitory PD-L1-targeted immunotherapy with chemotherapy (15). Notably, however, the involvement of the various types of soluble ICMs as potential contributors to systemic immune dysregulation in early BC remains largely unexplored, as does the role of NAC and subsequent surgical resection in alleviating cancer-related immune dysfunction.

In accordance with our primary hypothesis, we have therefore compared the plasma concentrations of eight co-stimulatory, six co-inhibitory, and two dual-active ICMs in a cohort of early BC patients with those of a group of healthy control subjects, as well as the effects of NAC and surgery on the plasma levels of these immunoregulatory proteins. This is a follow-up to our previous study in which we reported that the plasma levels of a number of systemic soluble co-stimulatory and co-inhibitory ICMs were significantly decreased in a cohort of early BC patients relative to those of healthy controls, indicative of tumor-related immune dysregulation (16).

## Patients and controls

Seventy-two female patients of the original cohort (n=98) (16) with early BC attending the Medical Oncology Centre of Rosebank, Johannesburg, South Africa, who had completed treatment (NAC) and surgery were deemed eligible for recruitment to the study. Eligibility criteria included: i) age ≥18 years; ii) Eastern Cooperative Oncology Group (ECOG) performance status (PS) of 0, 1, or 2; iii) histologically confirmed BC (17) classified as clinical stage I, II or III according to the AJCC Breast Staging 8th Edition (18); iv) normal bone marrow, liver and renal function; v) NAC with anthracycline (A) and/or a taxane (T)-based chemotherapy regimen. Human epidermal growth factor receptor 2 (Her2) -positive patients received neoadjuvant trastuzumab-based treatment.

Exclusion criteria were as follows: i) prior systemic chemotherapy for BC within five years; ii) a history of any other malignancy during the preceding five years, with the exception of basal cell or squamous cell carcinoma of the skin treated with local resection only, as well as carcinoma *in situ* of the cervix; iii) patients with confirmed stage IV disease; iv) known seropositivity for human immunodeficiency virus (HIV) and/or hepatitis B or hepatitis C viruses; v) uncontrolled intercurrent illness including, but not limited to, active infection, symptomatic congestive heart failure, unstable angina pectoris, cardiac arrhythmia or psychiatric illness/social situations that would limit compliance with study requirements; vi) pregnancy or breast feeding; and vii) otherwise not deemed to be a good study candidate according to the sole discretion of the principal investigator (BLR).

The group of healthy female control participants (n=45, mean age = 49.9 years; range 24–70 years) was recruited almost exclusively from the female personnel of the Medical Oncology Centre of Rosebank and the Faculty of Health Sciences, University of Pretoria. Exclusion criteria included: i) those with uncontrolled medical conditions; and ii) any potential participant deemed unwell by the qualified nursing sister in attendance on the day of venepuncture. Immunohistochemical staining was performed for ER, PR, Her2, and Ki67. Fluorescence *in situ* hybridization (FISH) was used to confirm Her2 positivity. Clinical assessment of the primary tumor and lymph nodes was done using bi-dimensional caliper measurements of the primary tumor and axillary nodes. The lymph node positivity at presentation was assessed clinically, radiologically, or by sentinel lymph node biopsy. Sonographic assessments of the primary tumor and lymph nodes were performed regularly. The pathological complete response (pCR) was defined as the complete disappearance of invasive cancer in the breast and the absence of tumor cells in the axillary lymph nodes.

## Ethics committee clearance

Permission to undertake this study and to draw blood from patients with early BC and from matched, healthy control subjects was granted by the Research Ethics Committee of the Faculty of Health Sciences, University of Pretoria, in full compliance with the World Medical Association Declaration of Helsinki 2013. Two submissions were approved: firstly, the BC study, and secondly, a submission in respect of the healthy control subjects (respective Approval Numbers 517/2017 and 762/2020). Prior, written informed consent was obtained from all participants.

## Methods

Whole blood samples of 20 mL and 10 mL volumes were collected in ethylenediaminetetraacetic acid (EDTA)-containing vacutainers from the BC patients and control subjects, respectively, and the plasma promptly separated, aliquoted and stored at -80°C until the plasma concentrations of the various soluble co-inhibitory and co-stimulatory immune checkpoints were measured.

### Soluble immune checkpoints

A Human Immuno-Oncology Checkpoint Protein Panel (Milliplex^®^ MAP Kit, Merck, KGaA, Darmstadt, Germany; catalogue number HCKP1-11K-PX17) was used to simultaneously determine the plasma concentrations of 16 soluble immune checkpoint molecules namely, cluster of differentiation (CD)27, CD28, CD40, CD80, CD86, glucocorticoid-induced tumor necrosis factor receptor family-related protein (GITR) and its ligand GITRL, inducible T cell costimulator (ICOS), B and T lymphocyte attenuator (BTLA), herpes virus entry mediator (HVEM), Toll-like receptor (TLR)-2, CTLA-4, PD-1, PD-L1, LAG-3 and TIM-3. All reagents were provided by the manufacturer and the experimental protocol was followed as per the manufacturer’s instructions. Briefly, plasma samples were thawed at room temperature and diluted at a ratio of 1:2 in assay buffer provided. Diluted plasma samples, standards and controls (25 μL) were added to the appropriately designated wells followed by the addition of the conjugated beads (25 μL). The plate was sealed with a foil cover and incubated for two hours at room temperature (25°C) with gentle agitation.

Following the incubation period, the plate was washed three times with 200 μL wash buffer using a Bio-Plex Pro Wash Station Magnetic Plate Washer (Bio-Rad Laboratories Inc., Hercules, CA, USA). Thereafter, 25 μL of detection antibodies were added to each well. The plate was sealed and incubated as described above for one hour at 25°C. This was followed by the addition of 25 μL streptavidin-phycoerythrin to each well. The plate was sealed again and incubated for a final 30 minutes as described above. The plate was then washed a further three times with 200 μL wash buffer. Sheath fluid (150 μL) was added to all wells and the beads were resuspended on a plate shaker for two minutes prior to being assayed on a Bio-Plex Suspension Array platform (Bio-Rad Laboratories Inc., Hercules, CA, USA). Bio-Plex Manager software 6.0 was used for bead acquisition and analysis of median fluorescence intensity. The results are reported as picograms (pg)/milliliter (mL) plasma.

### Pathological complete response

As mentioned above, the pCR was defined as the complete disappearance of invasive cancer in the breast and the absence of tumor in the axillary lymph nodes.

### Statistical analysis

The primary hypothesis was that significant differences exist in the plasma levels of soluble ICMs of newly diagnosed, early BC patients measured pre-treatment, post-NAC and post-surgery. Data, presented as the median values with 95% confidence intervals, was prospectively obtained and levels of ICMs measured at the different stages of treatment were compared in the cohort of BC patients, as well as with those of healthy controls using the non-parametric Mann-Whitney U-test. Descriptive statistics were used to tabulate patient characteristics. A *p* value of < 0.05 was considered statistically significant. The area under the receiver-operating curve (AUC) was used as a measure of the discriminatory abilities of the soluble ICMs. The Youden index, a summary measure of the receiver -operating curve (ROC), was used as an agnostic method for choosing an optimal cut-off value on the biomarker value to illustrate potential clinical usefulness. NCSS software version 11 for Windows (USA) was used for statistical analyses.

## Results

### Patient demographics, breast cancer subtypes

The plasma levels of soluble ICMs were measured in 72 patients with early BC receiving NAC. Patient characteristics are summarized in **Table 1**. The patients had a median age of 54 years (range 29 – 85 years), of which 63.9% were post-menopausal, 34.7% pre-menopausal and 1.4% peri-menopausal. The predominant biological subtype was triple -negative BC (TNBC) (70.8%), with the remaining subtypes being Her2- positive (13.9%), luminal-A (1.4%) and luminal-B (12.5%). One patient had TNBC and luminal-B (1.4%). The tumor size was classified as follows: T1 patients (29.2%), T2 patients (58.3%), T3 patients 8.3% and T4 patients 4.2%. Half of the patients had nodal involvement (50%).

**Table 1:** Demographic and clinical data of the cohort of newly diagnosed early breast cancer patients.

Patient Characteristics		
Age		
Median	54 years	
Range	29 - 85 years	
Menopausal Status		
Post-menopausal	46	63.89%
Pre-menopausal	25	34.72%
Peri-menopausal	1	1.39%
Grade		
1	1	1.39%
2	20	27.78%
3	49	68.06%
Unknown	2	2.78%
Tumor Size		
T1	21	29.17%
T2	42	58.33%
T3	6	8.33%
T4	3	4.17%
Nodal Status		
Positive	36	50.00%
Negative	36	50.00%
Stage		
1	12	16.67%
2A	32	44.44%
2B	20	27.78%
3	8	11.11%
Biological Type		
Her2 Positive	10	13.89%
Luminal A	1	1.39%
Luminal B	9	12.50%
TNBC	51	70.83%
TNBC & Luminal B	1	1.39%
Ki-67		
≤ 14%	3	4.17%
15 - 39%	23	31.94%
≥ 40%	45	62.50%
Unknown	1	1.39%

### Soluble immune checkpoints

Comparisons between the plasma concentrations of the soluble co-stimulatory, co-inhibitory and dual ICMs for: i) the cohort of BC patients prior to initiation of NAC relative to those of the healthy control subjects; and ii) pre-treatment versus post-NAC are shown in **Tables 2**, **3**, respectively, with the results expressed as the median values with 95% confidence limits. With respect to comparisons, the pre-treatment plasma concentrations of the soluble ICMs for the cohort of BC patients relative to those of the healthy control subjects, all of these were either significantly or numerically decreased, as reported previously (16). In the case of the eight soluble co-stimulatory ICMs, five of these, CD27, CD40, ICOS, GITR and GITRL, were significantly decreased (*p*<0.021-*p*<0.0001), while CD28, CD80 and CD86, were numerically decreased, but not significantly so (**Table 2**). Of the six co-inhibitory ICMs, three, namely CTLA-4, PD-L1 and TIM-3, were significantly decreased (*p*<0.008-*p*<0.00001) in the group of BC patients, while BTLA, LAG-3 and TIM-3 were numerically, but not significantly decreased. Of the two dual-active soluble ICMs, both HVEM and TLR-2 were lower in the group of BC patients, with only the former attaining statistical significance (*p*<0.0004).

**Table 2:** Comparison between newly diagnosed early breast cancer patients and control subjects with respect to the plasma concentrations of the soluble co-stimulatory, co-inhibitory and dual-activity immune checkpoints.

	ICM	Breast Cancer newly diagnosed (n=72)	Controls (n=45)	*p* value
		Median pg/mL (95%CI)	Median pg/mL (95%CI)	
**Co-stimulatory**	CD27	3342.45	4577.35	0.0243
		(2808.61 – 4107.68)	(3391.13 – 5784.85)	
	CD28	32914.45	46135.18	0.1248
		(29326.90 – 42636.04)	(27210.29 – 67544.10)	
	CD40	1523.32	1977.68	0.0210
		(1298.16 – 1777.45)	(1404.82 – 2569.56)	
	ICOS	15123.78	26506.65	0.0087
		(12471.47 – 19942.11)	(15897.52 – 31725.99)	
	GITR	1497.40	3797.68	0.0001
		(1053.33 – 1969.52)	(1993.96 – 5396.86)	
	GITRL	5886.13	7151.12	0.0199
		(4959.23 – 6681.22)	(5528.36 – 9878.41)	
	CD86	11585.17	14297.09	0.1734
		(9938.61 – 14646.29)	(9391.46 – 20525.14)	
	CD80	1678.33	2329.77	0.0735
		(1422.82 – 2039.65)	(1395.01 – 3042.87)	
**Co-inhibitory**	PD-1	12305.41	14917.48	0.5158
		(10260.08 – 15798.61)	(7874.92 – 21795.02)	
	PD-L1	1647.14	3342.62	0.0001
		(1269.54 – 2228.88)	(2628.64 – 4750.96)	
	CTLA-4	1566.38	2618.23	0.0079
		(1314.46 – 1890.81)	(1578.44 – 3110.47)	
	TIM-3	3897.66	5046.87	0.0005
		(3169.51 – 4330.5)	(4732.72 – 5958.87)	
	LAG-3	131275.90	150416.00	0.5868
		(106666.3 – 156881.5)	(94508.53 – 187997.2)	
	BTLA	13021.75	18147.26	0,2349
		(10277.82 – 18548.68)	(11461.86 – 25180.69)	
**Dual**	TLR-2	26831.35	30477.20	0,1806
		(21172.10 – 32396.97)	(20928.44 – 50302.64)	
	HVEM	1865.22	2290.19	0,0004
		(1671.15 – 2038.38)	(2079.46 – 2618.44)	

**Table 3:** Comparison of the concentrations of the soluble co-stimulatory, co-inhibitory and dual-activity immune checkpoints measured in the plasma of the cohort of early breast cancer patients before and after implementation of neoadjuvant chemotherapy (NAC).

	ICM	Breast Cancer newly diagnosed (n=72)	Post-NAC (n=72)	*p* value
		Median pg/ml (95%CI)	Median pg/ml (95%CI)	
**Co-stimulatory**	CD27	3342.45	5351.47	0.0001
		(2808.61 – 4107.68)	(4678.25 - 5894,.7)	
	CD28	32914.45	44277.76	0.0416
		(29326.90 – 42636.04)	(38319.44 – 51220.42)	
	CD40	1523.32	2030.72	0.0003
		(1298.16 – 1777.45)	(1792.5 – 2199.04)	
	ICOS	15123.78	26586.28	0.0002
		(12471.47 – 19942.11)	(20912.88 – 31335.04)	
	GITR	1497.40	4035.98	0.0001
		(1053.33 – 1969.52)	(3198.29 – 5204.35)	
	GITRL	5886.13	5339.99	0.8044
		(4959.23 – 6681.22)	(4728.24 – 6121.00)	
	CD86	11585.17	9922,61	0,2789
		(9938.61 – 14646.29)	(7890.94 – 11990.77)	
	CD80	1678.33	3048,74	0.0001
		(1422.82 – 2039.65)	(2522.82 – 3520.25)	
**Co-inhibitory**	PD-1	12305.41	13350.55	0.7859
		(10260.08 – 15798.61)	(10537.37 – 15491.33)	
	PD-L1	1647.14	4794.97	0.0001
		(1269.54 – 2228.88)	(4162.41 – 5731.71)	
	CTLA-4	1566.38	598,20	0.0001
		(1314.46 – 1890.81)	(472.91 – 768.78)	
	TIM-3	3897.66	9975.90	0.0001
		(3169.51 – 4330.5)	(8793.62 – 10515.70)	
	LAG-3	131275.90	464880.70	0.0001
		(106666.3 – 156881.5)	(309218.5 – 580137.6)	
	BTLA	13021.75	9987.98	0.0367
		(10277.82 – 18548.68)	(8255.35 – 12554.33)	
**Dual**	TLR-2	26831.35	33837.86	0.0258
		(21172.1 – 32396.97)	(28228.61 - 39571,02)	
	HVEM	1865.22	4047.29	0.0001
		(1671.15 – 2038.38)	(3610.92 – 4445.29)	

As shown in **Table 3**, following NAC, the plasma concentrations of six of the soluble co-stimulatory ICMs (CD27, CD28, CD40, CD 80, ICOS, and GITR increased significantly (*p*<0.04-*p*<0.00001) to levels comparable with those of the control group, while GITRL was essentially unchanged and CD86 numerically lower. Of the soluble co-inhibitory ICMs, the plasma concentrations of three of these molecules (PD-L1, LAG-3, TIM-3) increased significantly (all *p*<0.00001) (**Table 3**). Levels of PD-1 remained similar to pre-treatment levels, while those of BTLA and CTLA-4 were significantly lower (*p*<0.03 and *p*<0.00001, respectively) (**Table 3**). Of the two dual-active soluble ICMs, the plasma concentrations of TLR-2 and HVEM increased significantly post-NAC (*p*<0.02 and *p*<0.00001, respectively) relative to pre-treatment values.

Notably, as shown in **Table 4**, the post-NAC plasma levels of PD-L1 and those of LAG-3 and TIM-3 in particular, were also significantly higher than those of the control group (percentage increases of 43.5, 209% and 97.6% for PD-L1, LAG-3 and TIM-3, respectively), with the levels of HVEM also being significantly higher (77%) than those of the control group (**Table 4**).

**Table 4:** Comparison of the plasma concentrations of the soluble co-inhibitory immune checkpoints, PD-L1, LAG-3 and TIM-3 and the soluble dual-activity checkpoint, HVEM, measured in the group of healthy control subjects and the cohort of early breast cancer patients post-neoadjuvant chemotherapy (NAC).

Checkpoint (pg/ml)	Controls (n=45)	Breast cancer patients post-NAC (n=72)	*p* value
PD-L1	3343 (2268.64-4750.96)*	4795 (4162.41-5731.71)	0.0010
LAG-3	150416 (94508.3-187997.2)	464881 (309218.5-580137.6)	0.00001
TIM-3	5047 (4732.72-5958.87)	9976 (8793.62-10515.70)	0.00001
HVEM	2290 (2079.46-2618.44)	4047 (3610.92-4445.29)	0.00001

*Results are expressed as the median values (95% CI).

As shown in **Table 5**, on completion of NAC, no further statistically significant increases in the plasma concentrations of any of the 16 soluble ICMs were detected following surgical resection of residual tumor tissue. This observation seemingly indicates that NAC is the primary driver of alterations in the plasma levels of both co-stimulatory and co-inhibitory ICMs in our cohort of early BC patients.

**Table 5:** Comparison of the plasma concentrations of the soluble co-stimulatory, co-inhibitory and dual-activity immune checkpoints measured in the plasma of the cohort of early breast cancer patients post-NAC and post-surgery.

	ICM	Post-NAC (n=72)	Post-Surgery (n=72)	*p* value
		Median pg/mL (95%CI)	Median pg/mL (95%CI)	
**Co-stimulatory**	CD27	5351.47	5427.68	0.8105
		(4678.25 – 5894.37)	(4411.67 – 6317.06)	
	CD28	44277.76	50058.18	0.5705
		(38319.44 – 51220.42)	(34830.52 – 64706.44)	
	CD40	2030.72	2054.12	0.6203
		(1792.50 – 2199.04)	(1820.74 – 2383.79)	
	ICOS	26586.28	29746.46	0.3751
		(20912.88 – 31335.04)	(24270.58 – 33438.89)	
	GITR	4035.98	4434,89	0.9777
		(3198.29 – 5204.35)	(3354.37 – 6046.58)	
	GITRL	5339.99	5927.89	0.5226
		(4728.24 – 6121.00)	(4860.76 – 7008.59)	
	CD86	9922.61	12439.80	0.2276
		(7890.94 – 11990.77)	(9566.36 – 14837.24)	
	CD80	3048.74	3611.23	0.6459
		(2522.82 – 3520.25)	(2754.67 – 4138.41)	
**Co-inhibitory**	PD-1	13350.55	15076.64	0.3102
		(10537.37 – 15491.33)	(12077.71 – 19383.37)	
	PD-L1	4794.97	5215.05	0.8667
		(4162.41 – 5731.71)	(4239.88 – 5969.17)	
	CTLA-4	598.20	687.76	0.8292
		(472.91 – 768.78)	(550.45 – 828.91)	
	TIM-3	9975.90	9615.77	1.0000
		(8793.62 – 10515.70)	(8440.15 – 10984.92)	
	LAG-3	464880.70	500133.40	0.5992
		(309218.5 – 580137.6)	(466624.6 – 500748.6)	
	BTLA	9987.98	12777.20	0.3217
		(8255.35 – 12554.33)	(9507.29 – 15003.63)	
**Dual**	TLR-2	33837.86	37042.86	0.7782
		(28228.61 - 39571.02)	(29069.20 – 45880.29)	
	HVEM	4047.29	3950.36	0.6259
		(3610.92 – 4445.29)	(3611.92 – 4381.72)	

**Figure 1:**
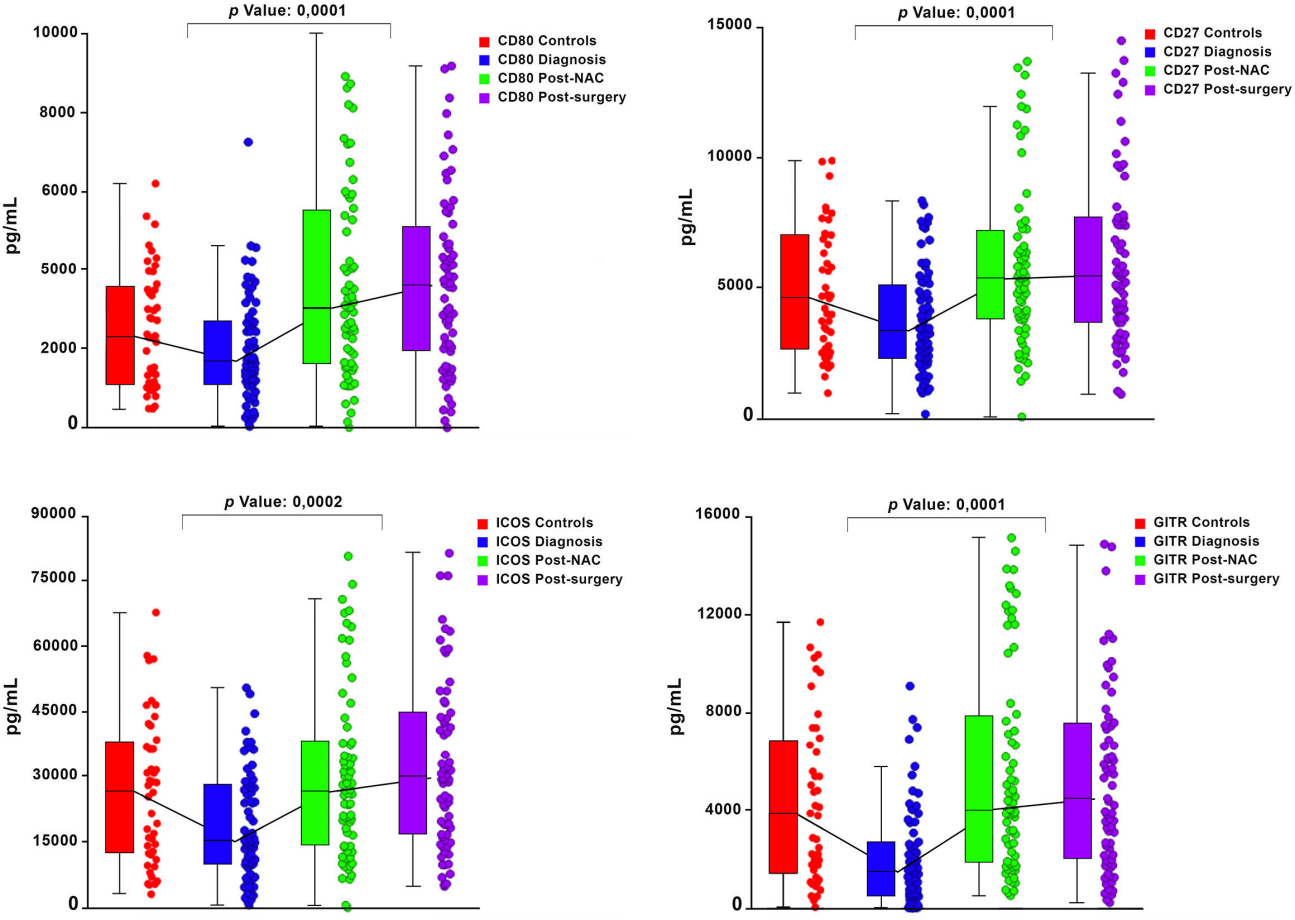
Box and whisker plots depicting the progressive changes in the median plasma concentrations (with 95% confidence limits) of four co-stimulatory immune checkpoints (CD27, CD80, ICOS and GITR) throughout the course of neoadjuvant chemotherapy (NAC) (pre-treatment/diagnosis, post-NAC and post-surgery) in relation to the corresponding median values of the control subjects. The *p* values represent the comparison between the pre-treatment/diagnosis and post-NAC values.

**Figure 2:**
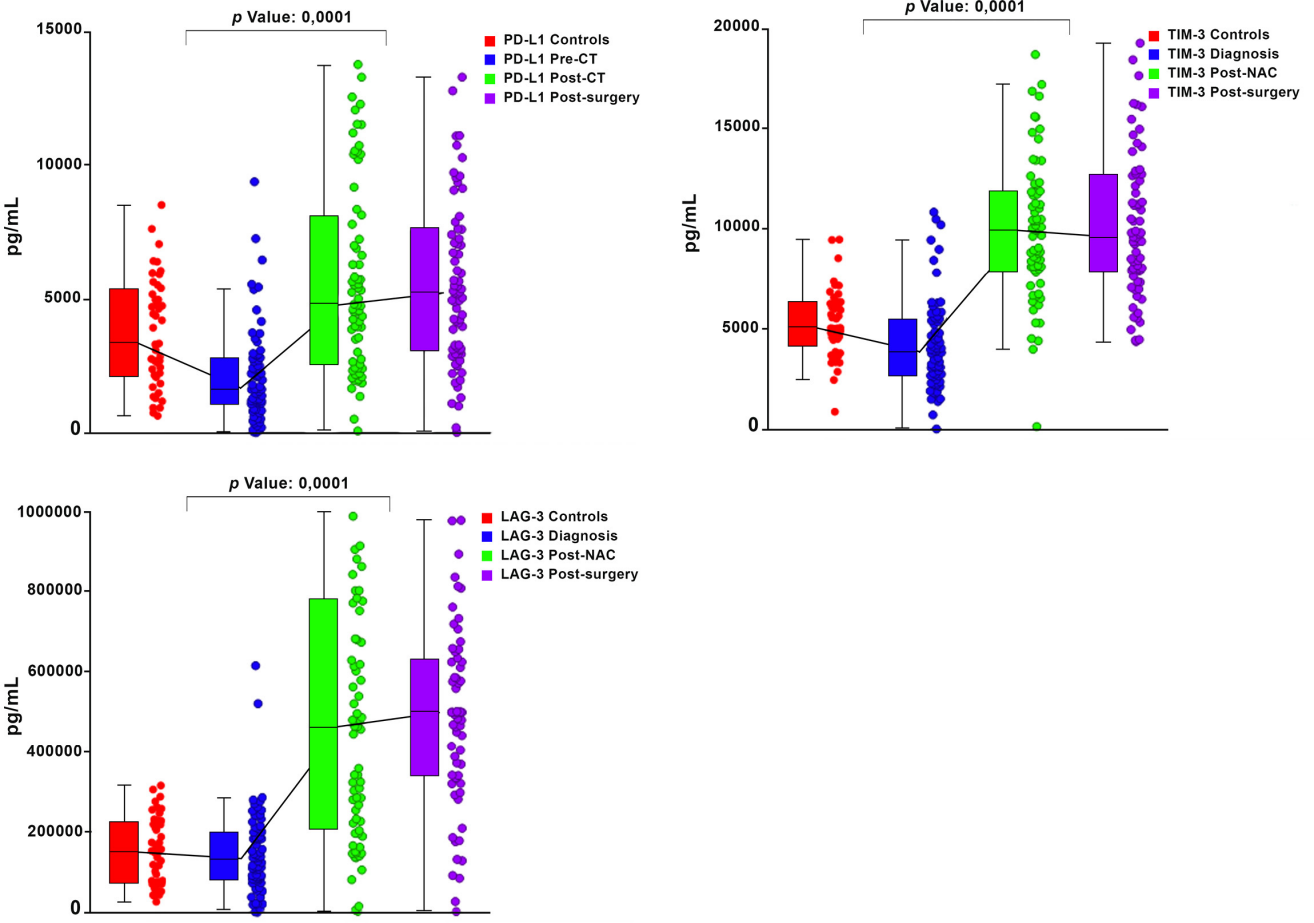
Box and whisker plots depicting the progressive changes in the median plasma concentrations (with 95% confidence limits) of three co-inhibitory immune checkpoints (PD-L1, TIM-3 and LAG-3) throughout the course of neoadjuvant chemotherapy (NAC) (pre-treatment/diagnosis, post-NAC and post-surgery) in relation to the corresponding median values of the control subjects. The *p* values represent the comparison between the pre-treatment/diagnosis and post-NAC values.

Graphical representations depicting the progressive changes in the median plasma concentrations of each of the 16 ICMs throughout the course of NAC (pre-treatment, post-NAC and post-surgery) in relation to the corresponding median values of the control subjects are shown in **Figure 1** (CD27, CD80, ICOS, GITR) and **Figure 2** (LAG-3, PD-L1, TIM-3), and **Supplementary Figure 1** (CD28, CD40, CD86, GITRL), **Supplementary Figure 2** (BTLA, CTLA-4, PD-1) and **Supplementary Figure 3** (TLR-2, HVEM).

### Response to neoadjuvant chemotherapy

Patients received taxane and/or anthracycline/alkylating agent-based NAC. Patients who tested positive for Her2 also received trastuzumab. There were 44 pCRs (61%) in the entire cohort. The pCR rates for Her2-positive disease, luminal disease and TNBC were 80%, 30% and 65%, respectively. An example of a patient who attained a pCR is shown in **Supplementary Figures 4–6**. None of the soluble ICMs was predictive or prognostic for pCR in this patient cohort. Patients were recruited between 24 Jan 2018 to 18 Sep 2019. Follow-up continued, and the database was updated for this analysis until January 2020. Five patients relapsed, and one died at a median follow-up of 364 days (79 to 643). The progression-free survival (PFS) and overall survival (OS) data will be reported separately at maturity.

## Discussion

In a forerunner to the current study, we recently reported that the plasma concentrations of a large group of soluble ICMs, comprising both co-stimulatory and co-inhibitory proteins, were significantly decreased in treatment-naïve, newly-diagnosed patients with early BC (n=98), relative to those of a cohort of healthy control subjects (n=45) (16). The current study is a follow-up to our earlier investigation and was designed to explore the immune-restorative potential of implementation of NAC and subsequent surgical resection of residual tumor tissue in the same, albeit somewhat smaller, cohort of early BC patients who completed both NAC and surgery (n=72). In terms of realization of our primary objectives, the following represent the most significant and novel findings: i) the plasma concentrations of five soluble co-stimulatory ICMs (CD27, CD40, GITRL, ICOS, GITR) were significantly lower than those of the control group pre-treatment, while those of CD28, CD80 and CD86 were numerically decreased, but not significantly so; ii) post-NAC, the plasma concentrations of six soluble co-stimulatory immune checkpoints (CD27, CD28, CD40, CD80, ICOS, GITR), all of which are involved in the activation of anti-tumor cytotoxic T cells (12), normalized; iii) recovery of the plasma levels of these six ICMs remained essentially unchanged following surgical resection; iv) the levels of three soluble co-inhibitory ICMs (PD-L1, LAG-3 and TIM-3) were also significantly lower in the cohort of early BC patients and increased significantly in magnitude relative to pre-treatment levels post-NAC, with no further augmentation post-surgery. Notably, and somewhat concerningly, the plasma levels of these three co-inhibitory ICMs were significantly higher, especially in the case of LAG-3 and TIM-3, than those of the control group.

Of the other soluble co-inhibitory ICMs, BTLA and CTLA-4 did not respond to NAC, remaining significantly decreased relative to their pre-treatment values, while the median level of soluble PD-1, which was comparable in both groups, remained unchanged. In the case of the persistently low levels of soluble CTLA-4, it is noteworthy that taxane has been reported to deplete the numbers of regulatory T cells (Tregs), a major cellular source of this ICM (19). However, further follow-up studies are necessary to establish if soluble CTLA-4 levels increase over time. Of the two dual-active soluble ICMs, the pre-treatment level of HVEM was significantly lower than that of the corresponding median control value, recovering post-NAC to values, which were considerably higher than those of the control group. In the case of soluble TLR-2, the median pre-treatment value for this dual-active ICM was comparable with that of the control group, increasing modestly, albeit significantly, post-NAC.

With respect to potential mechanisms that contribute to the increased systemic concentrations of the six soluble co-stimulatory ICMs following implementation of NAC in our cohort of early BC patients, it is noteworthy that the components of NAC, namely anthracycline, taxane, cyclophosphamide and platinum-based agents, are all effective inducers of immunogenic cell death, a process which restores anti-tumor immunity in the TME (20, 21). In this context, our findings are somewhat similar to a recently published study by Dong et al. (22). These authors reported that administration of the immunomodulatory agent, sorafenib, to patients with advanced hepatocellular carcinoma (n=53) resulted in significant increases in the median plasma concentrations of 11/16 of the soluble co-inhibitory and co-stimulatory ICMs tested, an effect which was evident after two weeks of therapy. Differences between our study and that of Dong et al., include the types of malignancy investigated, as well as types and duration of anticancer therapy; another notable difference between the two studies relates to the apparent absence of a group of healthy control subjects in the study reported by Dong et al. (22).

Given that the increases in the median plasma concentrations of the soluble co-stimulatory ICMs, together with associated clinical improvement in our BC patient cohort, may be linked to immunogenic cell death induced by NAC, the corresponding increases in the levels of the soluble co-inhibitory ICMs, PD-L1, LAG-3 and TIM-3, relative to those of the control group, do seem somewhat counterintuitive. Although this may relate to restoration of a balance in immune homeostasis, the unexpectedly high increases in the plasma levels of these co-inhibitory ICMs may, however, compromise the efficacy of therapeutic monoclonal antibodies (mAbs). In this context, it is noteworthy that persistently elevated plasma levels of soluble LAG-3 and TIM-3, like PD-L1, are associated with decreased survival in patients with various types of advanced malignancies (23–26).

Current approaches in the treatment of early BC include NAC, which in the case of TNBC in particular, may also incorporate PD-1/PD-L1 blockading mAbs as mentioned above (15). Against this background, two recently published, randomized, placebo-controlled clinical trials in early TNBC are noteworthy, namely the IMpassion031(n=333 patients) and KEYNOTE-522 (n=1174 patients) trials, which evaluated the clinical efficacy of addition of co-inhibitory ICM-targeted immunotherapy with either atezolizumab (PD-L1-targeted mAb) or pembrolizumab (PD-1-targeted mAb) in combination with NAC, relative to that of placebo + NAC, respectively (27, 28). The authors reported significantly improved pCR responses and event-free survival rates, irrespective of tumor PD-L1 positivity, in the Impassion031 and KEYNOTE-522 clinical trials, respectively (27, 28). These observations have been confirmed in two recent systematic reviews and meta-analyses. The first of these, which encompassed six randomized controlled trials focused on early TNBC (n=2142 patients), reported that NAC in combination with PD-1/PD-L1 inhibitors also resulted in improved pCR rates and event-free survival (29). The second included eight randomized controlled trials, encompassing 4901 patients with both early and metastatic TNBC (four trials in each category) (30). The authors of this study reported improved OS and PFS in patients treated with immunotherapy together with chemotherapy relative to those treated with chemotherapy alone (30). Taken together, these various studies are consistent with a beneficial, immune-restorative interaction between NAC and co-inhibitory ICM-targeted immunotherapy.

Although these and other studies document significant advances that have been made in treating TNBC patients (27–37), the magnitude of this benefit, as well as long-term survival, do, however, remain relatively low. Moreover, most of the clinical research in the field of immunotherapy has been focused on targeting PD-L1. Limitations of our study include sample size, diversity of BC subtypes and data collected from a single institution. Moreover, different therapeutic PD-1/PD-L1 therapeutic monoclonal antibodies require different companion tests. Some assays involve determination of PD-L1 levels on the tumor cells, some on immune cells and some on both cell types. An additional caveat is that only PD-L1 is extensively used in clinical practice. Problematically, however, some tumor types respond to immunotherapy independently of PD-L1 expression. Clearly, additional research of the type described in the current study, investigating additional checkpoints and additional pathways is required to improve long-term outcomes. These include discerning insights into the mechanisms of resistance against immune checkpoint blockade and the cell types involved, as well as the identification of novel, reliable targets and biomarkers to predict responsiveness to treatment. Importantly, the results of the present study indicate, that co-inhibitory ICMs other than PD-L1, specifically LAG-3 and TIM-3, are also prominent in early BC patients following implementation of NAC and should therefore be considered as potential targets for future investigation in these patients as an adjunctive strategy to improve outcomes.

## Conclusions

We previously described a state of systemic immune quiescence and immune dysregulation in patients with early BC, which was associated with decreased levels of soluble co-stimulatory and co-inhibitory ICMs. In the current follow-up study, we focused on the effect of NAC and surgery on the levels of these various ICMs. Novel findings originating from the current study include substantial increases in the levels of the majority of the soluble ICMs post-NAC, which did not change significantly post-surgery. Improvement in the levels of six of the co-stimulatory ICMs is indicative of immune- restoration, as all of these are involved in the activation of anti-tumor cytotoxic T cells. In the case of the co-inhibitory ICMs, we observed substantial increases in the levels of LAG-3, TIM-3 and PD-L1, identifying these ICMs as potential future targets in the management of TNBC. Although none of the 16 measured soluble plasma ICMs correlated with the pCR, an extended follow-up study involving a larger number of early BC patients is necessary to demonstrate possible associations of these soluble ICMs with OS and PFS.

## Data availability statement

The raw data supporting the conclusions of this article will be made available by the authors, without undue reservation.

## Ethics statement

Permission to undertake this study and to draw blood from patients with early BC and from matched, healthy control subjects was granted by the Research Ethics Committee of the Faculty of Health Sciences, University of Pretoria, in full compliance with the World Medical Association Declaration of Helsinki 2013. Two submissions were approved; firstly, the BC study, and secondly, a submission in respect of the healthy control subjects (respective Approval Numbers 517/2017 and 762/2020). Prior, written informed consent was obtained from all participants. The patients/participants provided their written informed consent to participate in this study.

## Author contributions

BR, HS, SN, and RA conceptualized the study. BR, CB, and SN provided clinical oversight. HS, AT, NH, TS, LK, and LH were responsible for diligent processing and storage of blood/plasma specimens and for performance of laboratory analyses of plasma specimens. BR, PM, and TS advised on aspects of statistical analysis of data. All authors contributed to the article and approved the submitted version.

## Supplementary material

Supplementary Figure 1 Box and whisker plots depicting the progressive changes in the median plasma concentrations (with 95% confidence limits) of three co-inhibitory immune checkpoints (BTLA, CTLA-4 and PD-1) throughout the course of neoadjuvant chemotherapy (NAC) (pre-treatment/diagnosis, post-NAC and post-surgery) in relation to the corresponding median values of the control subjects. The p values represent the comparison between pre-treatment/diagnosis and post-NAC values.

Supplementary Figure 2 Box and whisker plots depicting the progressive changes in the median plasma concentrations (with 95% confidence limits) of the remaining four co-stimulatory immune checkpoints (CD28, CD40, CD86 and GITRL) throughout the course of neoadjuvant chemotherapy (NAC) (pre-treatment/diagnosis, post-NAC and post-surgery) in relation to the corresponding median values of the control subjects. The p values represent the comparison between the pre-treatment/diagnosis and post-NAC values.

Supplementary Figure 3 Box and whisker plots depicting the progressive changes in the median plasma concentrations (with 95% confidence limits) of the two dual-activity immune checkpoints (TLR-2 and HVEM) throughout the course of neoadjuvant chemotherapy (NAC) (pre-treatment/diagnosis, post-NAC and post-surgery) in relation to the corresponding median values of the control subjects. The p values represent the comparison between the pre-treatment/diagnosis and post-NAC values.

Supplementary Figure 4 Histological photomicrographs of pre-treatment tissue of a patient who attained a pathological complete response. **(A)** x20 Magnification: Core biopsy hematoxylin and eosin (H&E) stained slide breast carcinoma no special type (NST), prior to therapy. **(B)** X10 Magnification: Positive E-Cadherin immunoperoxidase stain of tumor confirming ductal differentiation. **(C)** x20 Magnification: Estrogen receptor immunoperoxidase stain of tumor, showing no staining (ER negative).

Supplementary Figure 5 Histological photomicrographs of pre-treatment tissue of a patient who attained a pathological complete response. **(A)** x20 Magnification: Progesterone receptor immunoperoxidase stain of tumor (PR negative). **(B)** x20 Magnification: HER2 immunoperoxidase stain of tumor (HER2 negative). **(C)** x20 Magnification :Ki67 immunoperoxidase stain of tumor (90% of tumor cells staining positive).

Supplementary Figure 6 Histological photomicrographs of post-surgery tissue obtained during surgery of a patient who attained a pathological complete response. **(A)** X10 Magnification: Tumor bed post chemotherapy showing stromal fibrosis and dystrophic calcification with NO tumor cells H&E. **(B)** X10 Magnification: Tumor bed post chemotherapy showing loose fibrovascular response and elastosis with NO tumor cells H&E. **(C)** x20 Magnification: MNF116 (broad pancytokeratin) immunoperoxidase stain of tumor bed post chemotherapy showing NO residual staining tumor cells.
