# Supplementary figures and images for: Dysregulation of systemic soluble immune checkpoints in early breast cancer is attenuated following administration of neoadjuvant chemotherapy and is associated with recovery of CD27, CD28, CD40, CD80, ICOS and GITR and substantially increased levels of PD-L1, LAG-3 and TIM-3
Source: Front Oncol. 2023 Mar 30;13:1097309. doi: 10.3389/fonc.2023.1097309 (PMC10098332; doi:10.3389/fonc.2023.1097309)

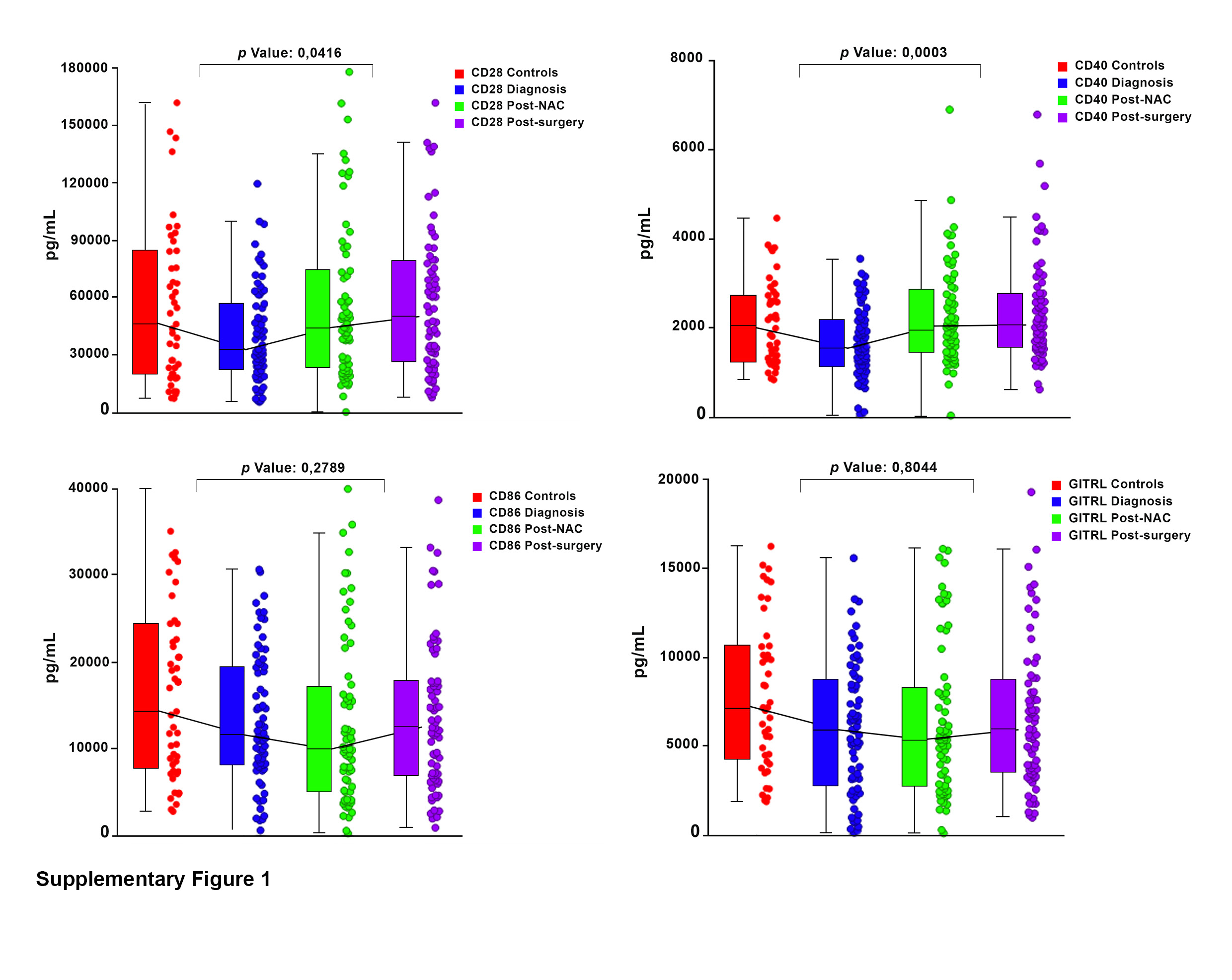

Supplement: Supplementary Figure 1 — Box and whisker plots depicting the progressive changes in the median plasma concentrations (with 95% confidence limits) of three co-inhibitory immune checkpoints (BTLA, CTLA-4 and PD-1) throughout the course of neoadjuvant chemotherapy (NAC) (pre-treatment/diagnosis, post-NAC and post-surgery) in relation to the corresponding median values of the control subjects. The p values represent the comparison between pre-treatment/diagnosis and post-NAC values. [file Image_1.jpeg]

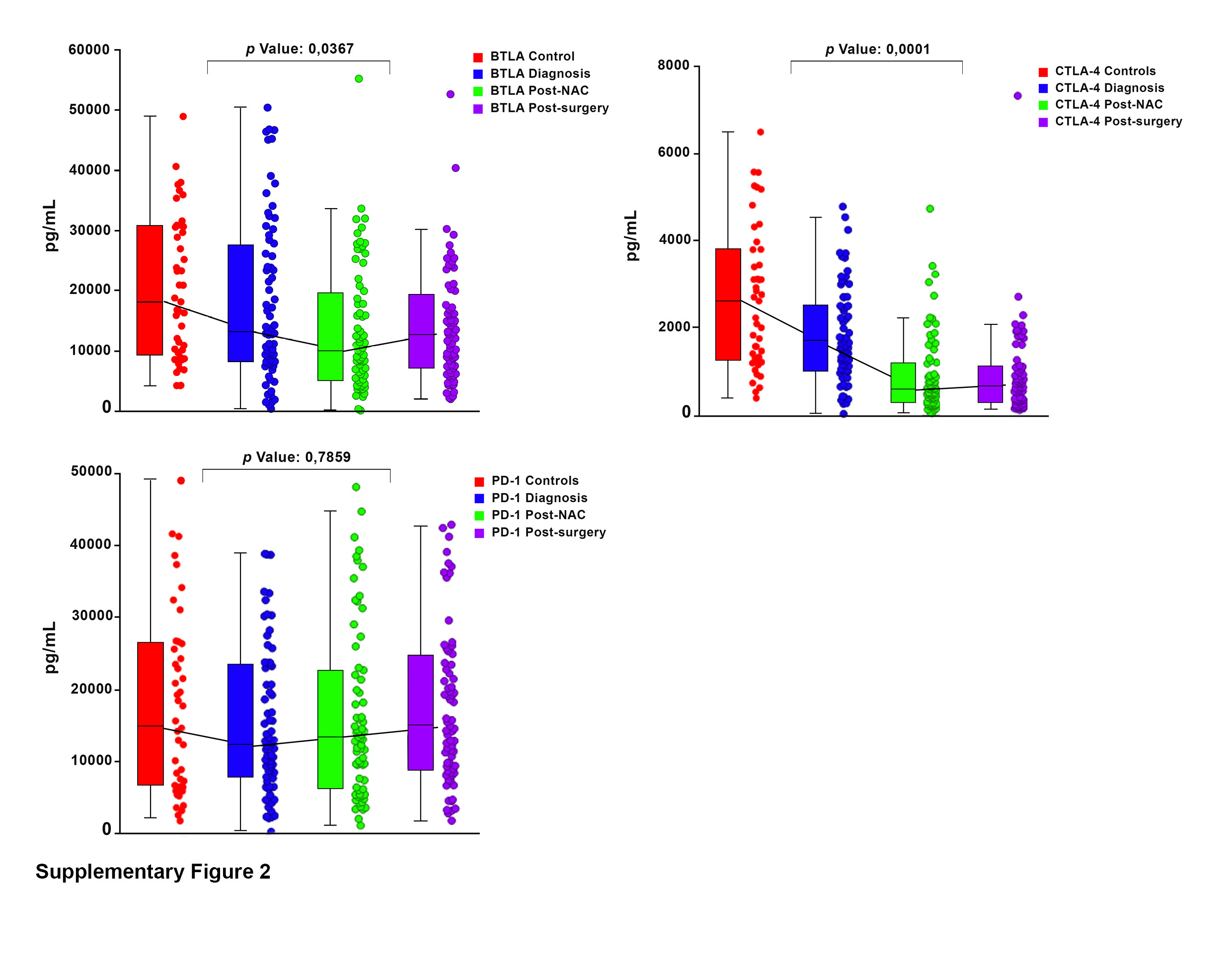

Supplement: Supplementary Figure 2 — Box and whisker plots depicting the progressive changes in the median plasma concentrations (with 95% confidence limits) of the remaining four co-stimulatory immune checkpoints (CD28, CD40, CD86 and GITRL) throughout the course of neoadjuvant chemotherapy (NAC) (pre-treatment/diagnosis, post-NAC and post-surgery) in relation to the corresponding median values of the control subjects. The p values represent the comparison between the pre-treatment/diagnosis and post-NAC values. [file Image_2.jpeg]

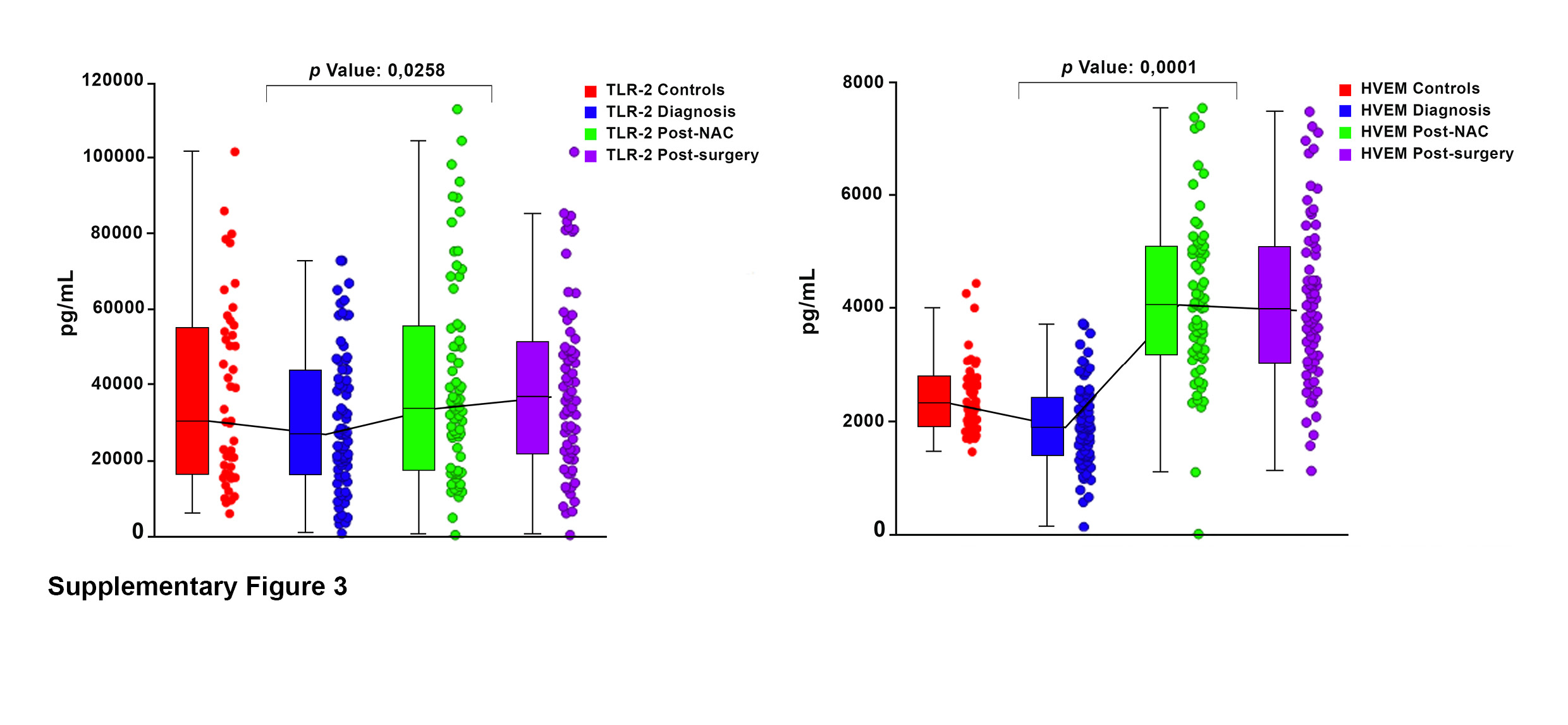

Supplement: Supplementary Figure 3 — Box and whisker plots depicting the progressive changes in the median plasma concentrations (with 95% confidence limits) of the two dual-activity immune checkpoints (TLR-2 and HVEM) throughout the course of neoadjuvant chemotherapy (NAC) (pre-treatment/diagnosis, post-NAC and post-surgery) in relation to the corresponding median values of the control subjects. The p values represent the comparison between the pre-treatment/diagnosis and post-NAC values. [file Image_3.jpeg]

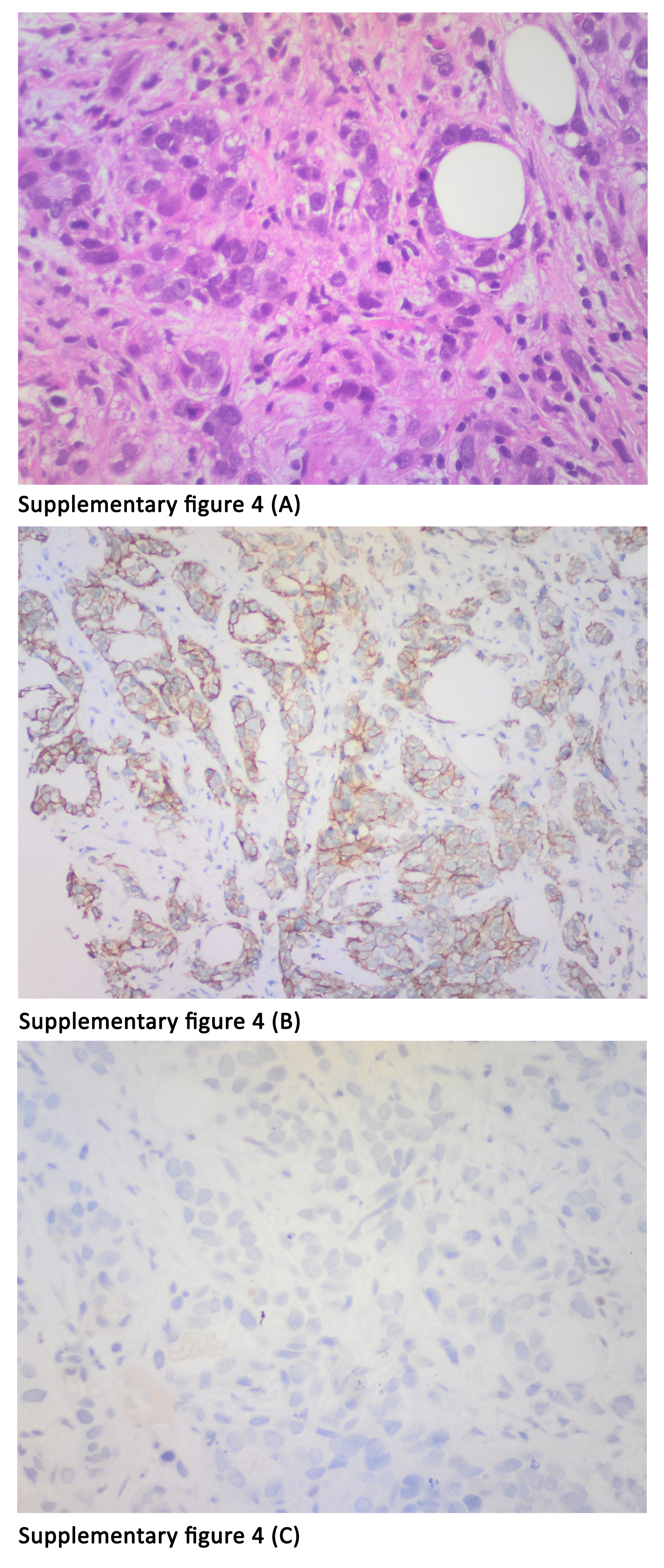

Supplement: Supplementary Figure 4 — Histological photomicrographs of pre-treatment tissue of a patient who attained a pathological complete response. (A) x20 Magnification: Core biopsy hematoxylin and eosin (H&E) stained slide breast carcinoma no special type (NST), prior to therapy. (B) X10 Magnification: Positive E-Cadherin immunoperoxidase stain of tumor confirming ductal differentiation. (C) x20 Magnification: Estrogen receptor immunoperoxidase stain of tumor, showing no staining (ER negative). [file Image_4.jpeg]

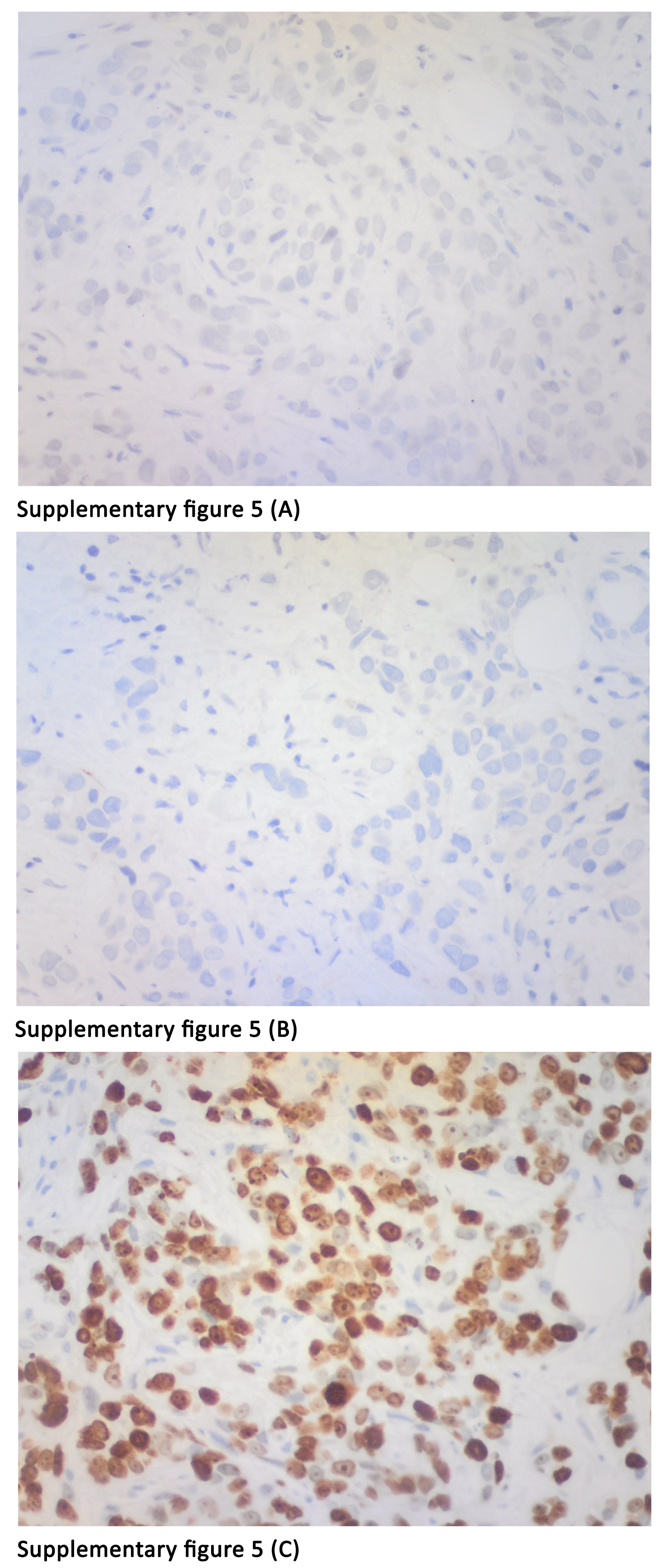

Supplement: Supplementary Figure 5 — Histological photomicrographs of pre-treatment tissue of a patient who attained a pathological complete response. (A) x20 Magnification: Progesterone receptor immunoperoxidase stain of tumor (PR negative). (B) x20 Magnification: HER2 immunoperoxidase stain of tumor (HER2 negative). (C) x20 Magnification :Ki67 immunoperoxidase stain of tumor (90% of tumor cells staining positive). [file Image_5.jpeg]

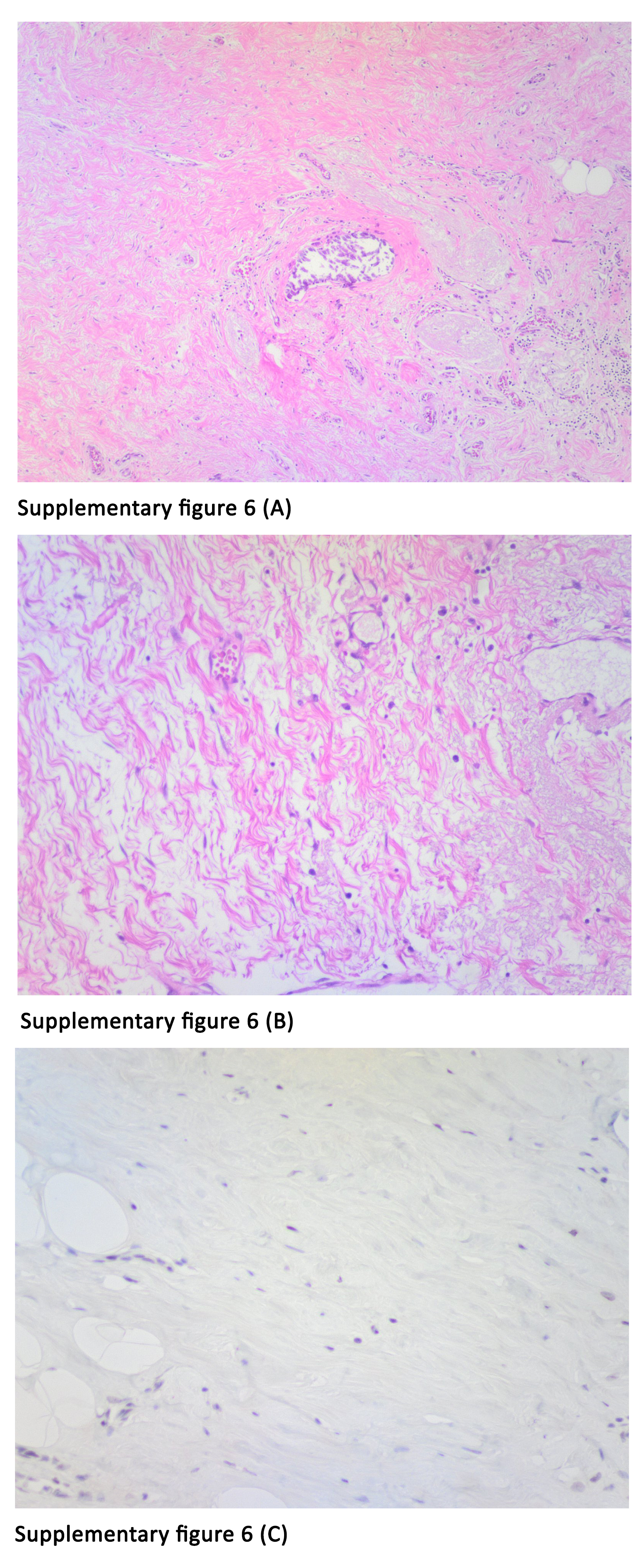

Supplement: Supplementary Figure 6 — Histological photomicrographs of post-surgery tissue obtained during surgery of a patient who attained a pathological complete response. (A) X10 Magnification: Tumor bed post chemotherapy showing stromal fibrosis and dystrophic calcification with NO tumor cells H&E. (B) X10 Magnification: Tumor bed post chemotherapy showing loose fibrovascular response and elastosis with NO tumor cells H&E. (C) x20 Magnification: MNF116 (broad pancytokeratin) immunoperoxidase stain of tumor bed post chemotherapy showing NO residual staining tumor cells. [file Image_6.jpeg]
